# Use of Probiotics in Intravaginal Sponges in Sheep: A Pilot Study

**DOI:** 10.3390/ani10040719

**Published:** 2020-04-20

**Authors:** Juan J. Quereda, Empar García-Roselló, Marta Barba, María L. Mocé, Jesús Gomis, Estrella Jiménez-Trigos, Esther Bataller, Rebeca Martínez-Boví, Ángel García-Muñoz, Ángel Gómez-Martín

**Affiliations:** Research Group Microbiological Agents Associated with Animal Reproduction (PROVAGINBIO), Department of Animal Production and Health, Veterinary Public Health and Food Science and Technology (PASAPTA) Facultad de Veterinaria, Universidad Cardenal Herrera-CEU, CEU Universities, 46113 Valencia, Spain; juan.quereda@uchceu.es (J.J.Q.); empar@uchceu.es (E.G.-R.); marta.barba@uchceu.es (M.B.); mmoce@uchceu.es (M.L.M.); jesus.gomis1@uchceu.es (J.G.); estrella.jimenez@uchceu.es (E.J.-T.); esther.bataller@uchceu.es (E.B.); rebeca.martinez@uchceu.es (R.M.-B.); angel@uchceu.es (Á.G.-M.)

**Keywords:** lactic acid bacteria, *Lactobacillus*, dysbiosis, alternative to antibiotics, vaginitis, estrus synchronization

## Abstract

**Simple Summary:**

The use of intravaginal devices may generate retention of vaginal secretions and abnormal discharges at device withdrawal, which may create changes in the normal vaginal bacterial microbiota. These changes in vaginal microbiota could allow the growth of opportunistic pathogenic bacteria which can require antibiotic treatment. The addition of antibiotics on sponges poses a risk of milk contamination. In dairy cows, it has been observed that the use of lactic acid bacteria (LAB) confers a health benefit to the reproductive tract against bacterial infections. However, LAB use in intravaginal sponges in sheep has not been reported. The aims of this study were to describe the dynamics of the ewes’s vaginal cultivable microbiota during a classic long-term estrous synchronization protocol and to develop a model of probiotic infusion in vaginal sponges in order to study their influence in ewe’s vaginal microbiota, general health status, fertility and prolificity. The present results suggest that LAB infusion in the ewe’s vagina do not affect general health status and did not reduce fertility during natural mating.

**Abstract:**

Sheep estrous synchronization is mainly based on progestagen-impregnated sponges which could cause vaginitis. Several species of *Lactobacillus* used as probiotics are commonly used in the treatment or prevention of urogenital infections in humans. However, no studies have been performed to analyze the potential use of probiotics to prevent urogenital infections in sheep. A randomized controlled clinical trial was conducted with 21 one-year-old ewes to develop a model of probiotic infusion in vaginal sponges in order to study their influence in ewe’s vaginal microbiota, general health status, fertility and prolificity. Synchronization of estrus was based on intravaginal sponges for 14 days. Bacterial communities (Enterobacteriaceae and lactic acid bacteria) were highly fluctuating over time and between animals. The safety of probiotic infusion (mix of *Lactobacillus* spp. 60% *L. crispatus*, 20% *L. brevis* and 20% *L. gasseri*) in the vagina of healthy ewes was firstly confirmed. Neutrophils were observed in 80% (8/10) of the control ewes compared to 36% (4/11) of the ewes in the probiotic group 2 days after sponge removal (*p* = 0.056). Fertility in the control and probiotic groups was 60% (6/10) and 91% (10/11), respectively *p* = 0.097. These results suggest that *Lactobacillus* spp. infusion in the ewe’s vagina does not affect general health status or fertility.

## 1. Introduction

Most protocols of estrous synchronization in ewes include the use of synthetic progestagens administered by intravaginal sponges for 12–14 days [1,2]. The use of intravaginal devices may generate retention of vaginal secretions and abnormal discharges at device withdrawal, which may create changes in the normal vaginal bacterial microbiota [3,4] and a lower fertilization rate [5,6,7,8]. These changes in vaginal microbiota could allow the growth of opportunistic pathogenic bacteria which can require antibiotic treatment [9,10]. The addition of antibiotics on sponges poses a risk of antibiotic residues presence in small ruminants milk [11]. Moreover, the emergence and spread of antimicrobial resistance underline the need for “antibiotic-free” strategies for disease control in veterinary medicine. Probiotic agents have been proposed as an alternative to antimicrobial compounds. Probiotics are live microorganisms, which confer a health benefit to the host when administered in adequate amounts (World Health Organization/Food and Agricultural Organization, 2001).

Vaginal microbiota establishes a mutually beneficial relationship with their host and plays a key role in health and disease [12]. Healthy human vaginal tract is predominantly colonized by different *Lactobacillus* species which play a key role in protecting women from genital infection [13]. Bacterial vaginosis in humans is characterized by the depletion of the normal *Lactobacillus*-dominant microbiota (e.g., *Lactobacillus crispatus* and *Lactobacillus jensenii*) and the overgrowth of commensal anaerobic bacteria. Bacterial vaginosis is then characterized by the reduction of the number of lactobacilli and an elevated vaginal pH [12,14]. Similar to fecal microbiota transplantation, vaginal microbiota transplantation is used to restore the mucosal microbiota imbalance by re-establishing beneficial vaginal microbial communities [15]. In dairy cows, it has been observed that the use of lactic acid bacteria (LAB) confers a health benefit to the reproductive tract against bacterial infections [16,17,18,19]. Little is known about the presence of LAB in ovine vaginal tract [20] and there is a lack of research regarding the utilization of probiotics during synchronization protocols to improve reproductive performance. The aims of this study were to describe the dynamics of the ewes’s vaginal cultivable microbiota during a classic long-term estrous synchronization protocol and to develop a model of probiotic infusion in vaginal sponges in order to study their influence in ewe’s vaginal microbiota, general health status, fertility and prolificity.

## 2. Materials and Methods 

### 2.1. Ethics Statement

All experimental procedures were approved by the University CEU Cardenal Herrera Care and Use Committee for Livestock and by the Spanish Regional Government Generalitat Valenciana (Animal use protocol 2018/VSC/PEA/0183). Ewes were cared for in accordance with the guidelines of the Spanish Policy for Animal Protection (RD53/2013) which complies with the European Union Directive 2010/63/UE about the protection of animals used for research.

### 2.2. Animals and Experimental Design

The study was conducted at University Research Farm (Náquera, Valencia, Spain latitude 39° N) from May to November. Twenty-one 1-year-old lambs (Romanov X Île de France) were included during the non-reproductive season. No signs of disease were observed in the animal population. Ewes were divided into two groups randomly. The control group (n = 10) was treated using the protocols established in commercial farms. One intravaginal progestagen-impregnated sponge (20 mg fluorogestone acetate, FGA, Chronogest^®^; MSD Animal Health, Madrid, Spain) was inserted for 14 days. The second group (probiotic; n = 11) was treated equally but infusing a lactic acid bacteria cocktail at the time of sponge application. At sponge removal, animals received one intramuscular injection of 350 IU of equine chorionic gonadotropin (eCG) (Foligon^®^, MSD Animal Health, Madrid, Spain). Previously to the application of the sponge, the vulva area was disinfected and all the material used for the application of the sponge was disinfected between animals. 

The experience began with the control group, followed a week later by probiotic group. Natural mating was used in order to remove the effect of the artificial insemination. After sampling the ewes for T-estrus, three males (Romanov X Île de France, 2-year-old) were introduced first in the control group and one week later in the probiotic group and were allowed to remain with the ewes for 4 days to test the fertility at the estrus synchronization and avoid possible estrus return. 

Fertility rate was assessed by transabdominal ultrasonographic examination (NanoMaxx, Sonosite, Bothell, WA, USA) 50 days after sponge removal and confirmed at lambing. Prolificity at parturition was also evaluated. 

### 2.3. Bacteria Isolation and Quantification

To investigate the effect of sponge and probiotic introduction in the vaginal tract, vaginal smears and vaginal swabs from synchronized ewes were obtained the following days:

- T0—The day of the sponge insertion.
- T-Estrus—Two days after sponge removal.
- T-Pregnancy—The day of pregnancy diagnosis, 50 days after sponge removal. At this time, only the ewes that were confirmed pregnant ultrasonographically were studied in order to have ewes with the same hormonal environment.

At T0, T-Estrus and T-Pregnancy, vulvar area was washed with chlorinated water, disinfected with neutral soap and then washed with water again. A sample from the deep vagina was taken from each ewe with sterile microbiological swabs (Deltalab, Eurotubo^®^, Barcelona, Spain) with transport media. All samples were collected using disposable gloves. The entire sample material was stored at 4 °C and transported to the laboratory within 1 h. In the laboratory, swabs were introduced in 10 mL sterile tubes containing 1 mL of Brain Heart Infusion media (Scharlab, Barcelona, Spain) and vortexed for 1 min. Serial dilutions were performed using PBS (Phosphate-Buffered Saline). Dilutions were plated in (1) MacConkey agar medium (Scharlab, Barcelona, Spain) for the selective growth and quantification of Enterobacteriaceae, (2) Man, Rogosa and Sharpe (MRS) (Scharlab, Barcelona, Spain) agar medium for the isolation of LAB and (3) sheep blood agar (Scharlab, Barcelona, Spain) as a general bacterial growth medium. Agar MacConkey and blood agar were incubated under aerobic conditions at 37 °C for at least 24 h. MRS were incubated under anaerobic conditions for 24 h at 37 °C. After incubation, bacterial colonies were counted and colony-forming units per mL (CFU/mL) were calculated.

### 2.4. Vaginal Microbial Diversity

Vaginal microbial diversity was assessed by quantifying the number of different bacterial colony types (distinctive cultivable phenotype) from vaginal swabs as previously reported by Rodriguez-Palacios et al., 2018 [21].

### 2.5. Vaginal Cytology

For cytological examination, the cotton swab was moistened with 0.2 mL of 0.9% saline solution and gently rolled onto a clean glass microscope slide and air-fixed. The smears were fixed with methanol for hematoxylin and eosin staining and were examined by a photomicroscope (Leica DM2000, Leica Microsistemas S.L.U., L’Hospitalet de Llobregat, Spain). The mean of 10 microscopic fields from each sample were evaluated at 400× magnification to identify and count inflammatory cells (neutrophils and macrophages) as previously described [3,22]. 

### 2.6. Probiotic Inoculation

The lyophilized commercial probiotic Femibiotic^®^ based on a mix of *Lactobacillus* spp. (60% *Lactobacillus crispatus*, 20% *Lactobacillus brevis* and 20% *Lactobacillus gasseri*) was reconstituted in enriched culture medium PPLO without antibiotic [23]. Probiotic concentration used in this preliminary in vivo study was based on previous studies. It has been described that commercial probiotics used in animals contain mixed LAB prepared at 1.7 × 10^4^ CFU/g and at 1 × 10^4^ CFU/g [24]. Taking into account these previous studies, two hundred µL with 3.24 × 10^6^ CFU/mL *Lactobacillus* spp. were inoculated intravaginally through a teflon catheter (BD Angiocath 14G 5.25in, ref. 382269) before sponge insertion in ewes of the probiotic group. The intrarectal temperature was evaluated at T0 and 24 h after probiotic inoculation.

### 2.7. Statistical Analysis

Presence or absence of neutrophils, Enterobacteriaceae, LAB, vaginal microbial diversity, fertility (pregnant or non-pregnant) and distribution of births (single or multiple) were analyzed using Chi-squared tests. CFU per mL were log transformed to normalize the data. Log CFU for Enterobacteriaceae and total number of bacteria and body temperature were analyzed with an ANOVA for repeated measures (mixed procedure). The animal was included as a random effect. The fixed effects included in the models were treatment (P and C) and time (T0, T-Estrus and T-Pregnancy) and vaginal microbial diversity (4 levels). Levene’s test for homoscedasticity was used to detect significant differences among group variances. In those cases, in which significant differences in the variances among studied groups were detected, a non-parametric test was used (Wilcoxon test) to test the differences between groups. Statistical analyses were performed using SPSS^®^ 24.0 (IBM Corporation, New York, NY, USA). All results were expressed as mean ± S.D. and the statistical significance was accepted at *p* < 0.05.

## 3. Results

### 3.1. Vaginal Microbiota and Cytology Changes after Intravaginal Sponge Removal in the Control and Probiotic Groups

**Figure 1:**
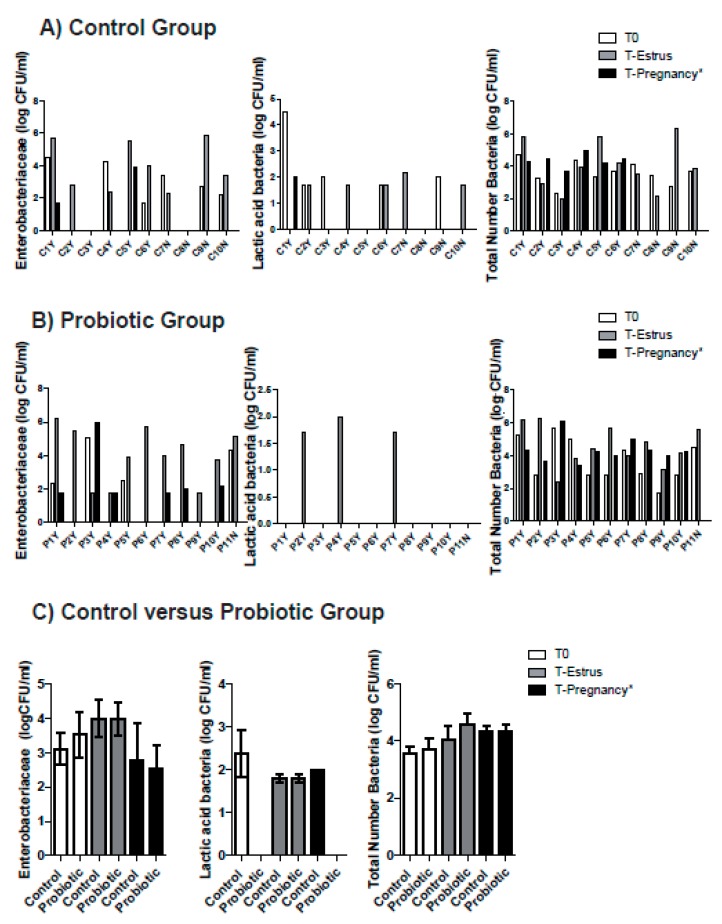
Number of Enterobacteriaceae (log CFU/mL), LAB (log CFU/mL) and total number of bacteria in the control (**A**), probiotic (**B**) and control versus probiotic groups (**C**) groups at T0, T-Estrus and T-Pregnancy. Pregnant animals: C1Y-C6Y and P1Y-P10Y and Non pregnant animals: C7N-C10N and P11N. * At T-Pregnancy only pregnant animals were studied.

In the control group, Enterobacteriaceae were observed in 60% (6/10) of the ewes at T0, 80% (8/10) at T-Estrus and 40% (2/5) at T-Pregnancy (non-significant differences) (Figure 1A) and no differences were observed in Enterobacteriaceae CFUs (Table 1). Two animals did not show Enterobacteriaceae at any time studied (Figure 1A, animal C3Y and C8N). LAB were observed in 50% (5/10) of the ewes at T0 and T-Estrus and 20% (1/5) at T-Pregnancy (non-significant differences) (Figure 1A). Some animals did not show LAB at any studied time (Figure 1A, animal C5Y and C8N) and no one showed LAB at the three times. For the total number of bacteria, significant differences between variances were detected, with T-Estrus showing a higher variance than T0 and T-Pregnancy (*p* < 0.05). Wilcoxon test did not detect significant differences between the three times (Table 1). 

In the probiotic group, Enterobacteriaceae were observed in 36% (4/11) of the ewes at T0, 100% (11/11) at T-Estrus and 60% (6/10) at T-Pregnancy (non-significant differences) (Figure 1B) and no differences were observed in Enterobacteriaceae CFUs (Table 1). LAB were observed in 0% of the ewes at T0 and T-Pregnancy, and in 27% (3/11) at T-Estrus (non-significant differences) (Figure 1B). Some animals did not show LAB at any studied time (Figure 1B, animals P1Y, P3Y, P5Y, P6Y, P8Y, P9Y, P10Y and P11N) and no one showed LAB at the three times.

No significant differences were observed for the total number of bacteria and Enterobacteriaceae between the control and probiotic groups at T0, T-Estrus and T-Pregnancy (Figure 1C and Table 1). At T-Estrus 80% of the ewes of the control group and 100% of the ewes of the probiotic group showed Enterobacteriaceae (non-significant differences). LAB were observed in 50% of the ewes in the control group and in 27% of the ewes in the probiotic group at T-Estrus (non-significant differences). No differences were found either for LAB at T-Pregnancy (20% of the ewes showed LAB in the control group and 0% in the probiotic group).

Most of the ewes showed mucopurulent vaginal discharge when intravaginal devices were removed (90% vs. 82% for control and probiotic group, respectively, no significant differences). Neutrophils were observed in 80% (8/10) of the ewes in the control group compared to 36% (4/11) of the ewes in the probiotic group on T-Estrus (*p* = 0.056). Macrophages were not observed at any time.

### 3.2. Probiotic Inoculation Effect on General Health Status, Fertility and Prolificity

General health status of ewes was not affected, and no hyperthermia was observed 24 h after *Lactobacillus* spp. intravaginal inoculation in the probiotic group (T0 39.3 °C ± 0.11 vs. T24 h 39.3 °C ± 0.11). Assessment of fertility of sponge-induced estrus did not show significant differences between control and probiotic groups (60% vs. 91% pregnant ewes, for control and probiotic group respectively, *p* = 0.097). The distribution of ewes with single and multiple births was similar with a mean litter size of 2.17 ± 0.41 for the control group and 1.60 ± 0.52 for the probiotic group. Multiple births were observed in 100% of the ewes of the control group and 60% of the ewes of the probiotic group (non-significant differences). 

### 3.3. Vaginal Microbial Diversity is Related to Enterobacteriaceae Counts 

**Figure 2:**
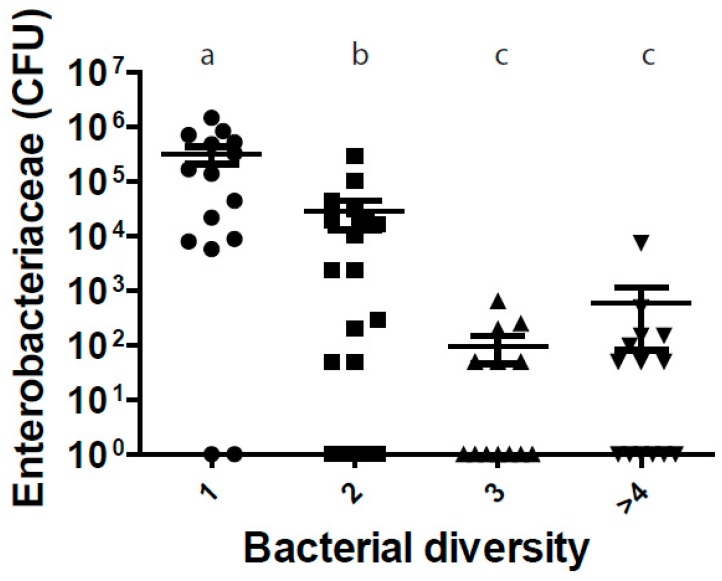
Relationship between the quantity of Enterobacteriaceae and the vaginal bacterial diversity of all ewes used in the study (control and probiotic group). Each dot represents the value for one ewe. Columns showing different letters (a, b, c) represent values significantly different from each other (*p* < 0.05).

In order to investigate whether vaginal microbial diversity is related to vaginal colonization by Enterobacteriaceae, we plotted together the quantity of Enterobacteriaceae and the vaginal bacterial diversity of all ewes used in the study (control and probiotic group). Vaginal tracts of the ewes with higher microbial diversity had lower Enterobacteriaceae counts (*p* < 0.05, Figure 2).

## 4. Discussion

This preliminary study showed that probiotic infused together with vaginal sponges did not affect general health status and did not interfere in ovine fertility during natural mating. To the best of our knowledge, there are no studies concerning the use of probiotics in the ewe’s reproductive tract. We first report the dynamics of the ewe’s vaginal cultivable microbiota (Enterobacteriaceae, LAB and total numbers of bacteria CFU) before intravaginal insertion of sponges (T0), T-Estrus and T-Pregnancy diagnosis. We describe that Enterobacteriaceae are frequently isolated in the vaginal tract of 1-year old ewes. Enterobacteriaceae in the vaginal tract may come from the gastrointestinal tract due to the anatomy of the ewe’s reproductive tract as described by other authors in the cow [25,26]. Here, we described that LAB CFUs are very scarce in the ewe’s vagina independently of the time evaluated as previous studies reported using culture-independent method (16S rRNA sequencing) in ruminants [20,27]. Bacterial communities studied (Enterobacteriaceae and LAB) were highly fluctuating over time and between animals, as previously described by other authors in bovine uterus [28,29].

In the present study, no significant differences were observed in the total number of bacteria on T0 and on T-Estrus, but in the control group variance was higher during T-Estrus, which could suggest that intravaginal sponge altered the ewe’s vaginal ecosystem dynamics and could increase instability during fertilization process. These results are in agreement with previous studies showing that intravaginal sponges are associated with abnormal discharges and vaginitis at sponge withdrawal [8]. Changes in the vaginal microbiota after long term progestagen treatment predispose to inflammatory and infectious processes which result in abnormal vaginal discharges [30]. Moreover, intravaginal sponges have been shown to generate histological and cytological alterations at withdrawal (hyperplasia, hypertrophy, hemorrhage and perivascular infiltrate and augmented numbers of neutrophils, macrophages and erythrocytes) [3].

Lactobacilli administered in the vagina reduced the occurrence of vaginal infections in women and lowered the incidence of uterine infections in periparturient dairy cows [16,31]. Bovine vaginal lactobacilli are able to inhibit specific metritis pathogens [32]. Vaginitis problems associated with the use of sponges led us to study the probiotic effect of *Lactobacillus* spp. in the vaginal tract of ewes. Our preliminary results showed some indications that *Lactobacillus* spp. could have some positive effects (i.e., close to statistical significant reduction of vaginal inflammation) without indications of adverse effects (it did not affect general health status, and did not reduce fertility). The lack of statistically significant differences could be due to the relatively small sample size used in the current study, suggesting that a larger study will be necessary to have more statistical power.

Since the loss of microbiota diversity is a constant finding of gut dysbiosis and disease [33], we investigated whether vaginal microbial diversity is related to vaginal colonization by Enterobacteriaceae (which are a common cause of vaginal infection). In our experimental conditions, vaginal microbial diversity was related to Enterobacteriaceae counts since the vaginal tracts of the ewes with higher vaginal microbial diversity had lower Enterobacteriaceae counts. These results are in agreement with Miranda-CasoLuengo (2019) [34] who showed that in cows reduced bacterial diversity in the vaginal microbiome was associated with later development of postpartum endometritis.

Intravaginal sponges lead to altered ewe’s vaginal ecosystem and increased instability at fertilization time (two days after sponge withdrawal). To the best of our knowledge, we report for the first-time the use of LAB to prevent urogenital infections in sheep. Our results suggest that probiotic use during the application of vaginal sponges could represent a new alternative strategy to the use of antibiotics during the reproductive protocols in small ruminants. Future investigations could be based on the current study where we inoculated a liquid LAB culture intravaginally through a catheter before sponge insertion in ewes.

## 5. Conclusions

In the current study, we report that Enterobacteriaceae and LAB vaginal populations were highly fluctuating over time and between animals during a classic long-term estrous synchronization protocol. We also report for the first-time the use of probiotics to prevent urogenital infections in sheep. Furthermore, our results showed some indications that LAB infusion in the ewe’s vagina could produce positive effects without indications of adverse effects (it did not affect general health status, and did not reduce fertility). A larger trial would be necessary to confirm the results of the present study.

## Figures and Tables

**Table 1:** Number of Enterobacteriaceae (log CFU/mL) and total number of bacteria (log CFU/mL) at T-Estrus and T-Pregnancy in control and probiotic group.

Time	Bacterial Isolation	Control Group	Probiotic Group
T0	Enterobacteriaceae	3.11 ± 1.12	3.29 ± 1.35
	Total Number of Bacteria	3.56 ± 0.72	3.68 ± 1.30
T-Estrus	Enterobacteriaceae	4 ± 1.31	4 ± 1.66
	Total Number of Bacteria	4.05 ± 1.53	4.58 ± 1.26
T-Pregnancy	Enterobacteriaceae	2.79 ± 1.54	2.53 ± 1.67
	Total Number of Bacteria	4.32 ± 0.48	4.34 ± 0.76
